# Technology-Enhanced Consultations in Diabetes, Cancer, and Heart Failure: Protocol for the Qualitative Analysis of Remote Consultations (QuARC) Project

**DOI:** 10.2196/10913

**Published:** 2018-07-31

**Authors:** Sara E Shaw, Deborah Cameron, Joseph Wherton, Lucas M Seuren, Shanti Vijayaraghavan, Satyajit Bhattacharya, Christine A’Court, Joanne Morris, Trisha Greenhalgh

**Affiliations:** ^1^ Nuffield Department of Primary Care Health Sciences University of Oxford Oxford United Kingdom; ^2^ Faculty of Linguistics University of Oxford Oxford United Kingdom; ^3^ Barts Health NHS Trust London United Kingdom

**Keywords:** remote consultations, communication, language, linguistics, cancer, diabetes mellitus, heart failure

## Abstract

**Background:**

Remote videoconsulting is promoted by policy makers as a way of delivering health care efficiently to an aging population with rising rates of chronic illness. As a radically new service model, it brings operational and interactional challenges in using digital technologies. In-depth research on this dynamic is needed before remote consultations are introduced more widely.

**Objective:**

The objective of this study will be to identify and analyze the communication strategies through which remote consultations are accomplished and to guide patients and clinicians to improve the communicative quality of remote consultations.

**Methods:**

In previous research, we collected and analyzed two separate datasets of remote consultations in a National Institute for Health Research–funded study of clinics in East London using Skype and a Wellcome Trust–funded study of specialist community heart failure teams in Oxford using Skype or FaceTime. The Qualitative Analysis of Remote Consultations (QuARC) study will combine datasets and undertake detailed interactional microanalysis of up to 40 remote consultations undertaken by senior and junior doctors and nurse specialists, including consultations with adults with diabetes, women who have diabetes during pregnancy, people consulting for postoperative cancer surgery and community-based patients having routine heart failure reviews along with up to 25 comparable face-to-face consultations. Drawing on established techniques (eg, conversation analysis), analysis will examine the contextual features in remote consultations (eg, restricted visual field) combined with close analysis of different modes of communication (eg, speech, gesture, and gaze).

**Results:**

Our findings will address the current gap in knowledge about how technology shapes the fine detail of communication in remote consultations. Alongside academic outputs, findings will inform the coproduction of information and guidance about communication strategies to support successful remote consultations.

**Conclusions:**

Identifying the communication strategies through which remote consultations are accomplished and producing guidance for patients and clinicians about how to use this kind of technology successfully in consultations is an important and timely goal because roll out of remote consultations is planned across the National Health Service.

**Registered Report Identifier:**

RR1-10.2196/10913

## Introduction

### Background

Health services face rising costs because of increasing disease prevalence, high “did not attend” (DNA) rates, and poor patient engagement, resulting in poor health outcomes and greater use of emergency care [1,2]. Most outpatient models fail to reliably provide responsive care when patients need intervention. The search is on for new and affordable ways of delivering care, particularly for those with chronic illnesses. Current national-level policy places considerable faith in digital technologies and their potential to deliver more efficient, effective, and patient-centric care [3-6]. Digital technology plays a significant, though varied, role in health system plans in reconfiguring hospital services and transforming the delivery of health services [7]. Attending regular clinics can be expensive, physically challenging, and inconvenient for patients [8]. Remote consultations (using Skype or similar applications) have the potential to fundamentally change the way in which patients interact with clinicians. However, the Web-based environment is known to produce subtle alterations in the dynamics of human interaction, potentially increasing the risk that clinical clues will be missed or the clinician-patient dynamic will be altered adversely [9,10]. As a radically new service model, it also carries operational and interactional challenges, including providing technical support, training staff and patients in using digital technologies, and avoiding potential for misunderstandings when (potentially sensitive) information is transferred remotely.

The current evidence base on remote consultations is sparse but has begun to develop [11-20]. A 2015 review identified 27 published studies that used Skype and similar technologies in clinical care, all but one of which reported positive benefits [12]. Most of these studies, and those published since [8,17,21-24], are brief descriptions of small pilot-stage projects or use experimental methods, especially randomized controlled trials (RCTs), to compare the remote option with traditional face-to-face encounters. Many of these studies focus on the use of Skype to support remote consulting with fewer examining other options, for example, FaceTime, Whatsapp, or purpose built applications [25]. A small number of studies examine combinations of technologies, for example, use of Skype plus texting [26] or use of remote consultations plus monitoring [27]. However, despite reported benefits, for example, in terms of increased access for patients [9,28-30], particularly those with complex needs [31], patient and clinician satisfaction with the remote option [23,32-35], potential time and cost savings [8,36,37], improvement in self-management skills [38], and improved compliance to treatment and/or clinical outcomes [17,22,24,31,39-43], small sample sizes (eg, 5 patients), and high losses to follow-up prevent any unqualified conclusions that remote consultations are “effective.”

In many published studies, technical and communication issues are mentioned but are not explored in depth. A number of studies have focused on the patient-provider relationship and concluded that there is little, if any, difference when comparing remote consultations with usual face-to-face care [44]. One study focused on the strength of the relationship among patients, caregivers, and health care professionals when behavioral health care was provided for adolescents with poorly controlled type 1 diabetes mellitus [45]. They concluded that the therapeutic relationship was similar to clinic-based care (on the basis of both adolescent and parent reporting via a survey) and that the relationship or care provided was largely unaffected by remote consulting. The research did not include close examination of communication or the role of technology.

There is recognition in the literature of the potential effects of remote consulting on satisfaction, adherence and compliance, health and clinical status, recall and understanding, and psychological well-being in the context of health care consultations [46,47]. There is currently limited published research that explores such potential effects. There is extensive evidence focusing on communication and interaction in health care consultations, highlighting how communication is shaped by wide ranging factors, such as patient preferences and available time [48-50], patient and clinician ethnicity, gender, behavior, and orientation to patient-centered care [50-53], interpretation (eg, of parental requests for further information) [54], nonverbal communication [55], and the use of technology (eg, electronic patient records) [56-58]. To our knowledge, there are no studies reporting the impact of remote consulting technology on communication and interaction in medical consultations. We found 2 studies that examined the quality of communication in the context of telemedicine consultations, one with primary care providers and patients consulting with specialists across a range of conditions using modular video/audio systems at either end [59] and the other with older patients requiring pulmonary medicine consultations and using a live 2-way audio and videoconferencing service [60]. Findings from both papers suggested that the use of telemedicine influences communication with doctors more likely to dominate telemedicine consultations. To our knowledge, there have been no papers examining the quality of communication in the context of Skype or similar Web-based media. Studies beyond the medical literature highlight the ways in which such media might alter interaction, for instance, by subtly desynchronizing communication [61,62]. There are questions about whether technical failures (eg, connecting but hearing no sound), new communicative foci (eg, “talking heads,” showing digital objects), new types of greetings (eg, the opening sequence of a video meeting), or interruption (eg, a family member entering the room) impact the consultation [62-68]. This evidence has yet to be considered in relation to remote medical consultations. We are still unaware how different communication strategies, modes of communication (speech, bodily conduct, gaze, and posture), and/or the material properties of the technology shape and constrain interactions in remote consultations.

### Our Research on Remote Consultations to Date

The Qualitative Analysis of Remote Consultations (QuARC) project, which is described in this paper, builds on previous research by our team, especially the Diabetes, Review, Engagement and Management via Skype (DREAMS) study, funded by the Health Foundation from 2012 to 2014, the Virtual Online Consultations-Advantages and Limitations (VOCAL) study, funded by the National Institute for Health Research (NIHR) Health Services and Delivery Research program from 2015 to 2017, and the Oxford Telehealth Qualitative Study (OTQS), funded by the Welcome Trust as part of a wider program of research undertaking Studies of Co-creating Assisted Living Solutions from 2015 to 2020.

VOCAL was a multilevel qualitative study on remote (“Skype” and similar) consultations involving macro level data (on national policy and industry strategy relating to remote consultations), meso level data (on organizational tasks and processes), and micro level data (videotaped consultations). Combined with the findings of DREAMS (an earlier, smaller study in the same setting), these findings showed that remote consultations appeared safe, effective, and convenient for patients who were preselected by their clinicians as “suitable” (although such patients represent a small fraction of clinic workloads) and were associated with improved DNA rates, reduction in Accident & Emergency attendance, improvements in blood glucose control, increased patient satisfaction, and lower patient-borne costs [19,69].

OTQS is a qualitative case study exploring telehealth and videoconsulting in patients with heart failure in the context of a large, UK-wide RCT. Study results indicate that most patients are judged “unsuitable” for remote consultations by clinicians or preferred to be seen face-to-face (in part because patients with heart failure have frequent comorbidities and are often frail, making their care complex and the course of their condition unpredictable). Despite these issues, there are remote consultations that patients and clinicians describe as “successful,” in which much of the focus is on lifestyle aspects of the condition (eg, questions about exercise tolerance and sleep quality, which indicate both physiological status and functional consequences) and medication compliance. We have observed successful discussions about medication, including a nurse identifying and correcting a misunderstanding of what dose of medication to take. We have also observed heart failure nurses successfully talking patients and/or relatives through self-examination of ankle edema and blood oxygen and blood pressure testing.

The combined dataset obtained from VOCAL and OTQS recordings offers opportunities for addressing questions about communication and quality of care in remote consultations. Preliminary analysis of the videos and transcripts across both studies suggests that remote consultations have advantages (eg, patients generally feel satisfied and many prefer consulting from the comfort of their own homes with family around them; clinicians who regularly use Skype or similar media are keen on this medium) but that they are different (eg, compared with the equivalent face-to-face encounter the overall length is shorter), even when taking account of the small amount of “technical talk” at the beginning (eg, “can you see me?” or “is the video on?”), and the flow of conversation is less natural [18]. In remote and face-to-face consultations, clinicians did more talking and exerted more control. One difference that was statistically (and probably clinically) significant was that both parties sometimes needed to state things explicitly in a remote consultation that remained implicit (and/or obvious to both parties) in a traditional face-to-face encounter. We also observed several examples of technical failure, including human error (eg, forgetting passwords) that significantly interfered with the quality of the consultation, with patients or staff not always sufficiently skilled or confident to undertake the necessary “troubleshooting” to achieve and maintain the video connection. More detailed methods and analysis from the VOCAL study can be found in the study report [70] and main findings paper [18].

To summarize, the existing evidence suggests that there is great potential for the use of Web-based media tools, such as Skype, for remote communication between patients and clinicians. However, while studies are broadly positive, the select nature of samples, small sample sizes, and high losses to follow-up raise questions about conclusions that the technology is “effective.” Literature, specifically on remote consultations, is currently limited. The contribution of Web-based media to consultations in health care has been studied mainly using experimental methods, especially RCTs, which have generally focused on evaluating the outcomes of the technology. There is extensive evidence focusing on the communication and interactions in medical encounters, for example, Stivers and colleagues [71], Stevenson [50] and Robinson [72], but none that examines the detail of interaction when consultations take place remotely. Evidence from studies beyond the medical literature highlighting the ways in which Skype and similar media might alter interaction (eg, desynchronizing communication) has yet to be considered in relation to remote consultations. In short, there is a significant knowledge gap in relation to the fine detail of communication in remote consultations. Addressing this gap and producing guidance for patients and clinicians about how to use this kind of technology successfully in consultations is an important and timely goal because roll out of remote consultations is planned across the National Health Service (NHS).

## Methods

### Aims

To identify and analyze the communication strategies through which remote consultations are accomplished and produce guidance for both patients and clinicians for improving the communicative quality of remote consultations.

### Objectives

Our objectives are as follows:

1. To analyze a multimodal dataset of up to 40 remote consultations with patients diagnosed with diabetes, cancer, and heart failure, and their clinicians (and compare these with a subset of up to 25 audio-recorded face-to-face consultations) using a combination of ethnographic and microanalytic approaches to investigate, in detail, how interactions are affected by mediation via Skype or similar applications
2. To generate findings on the detailed dynamics of communication and interactions in remote consultations and bring patients and clinicians, who have been involved in remote consultations together for a consolidating learning workshop to gather feedback and develop/refine resources
3. To develop provisional guidance for patients and clinicians on conducting remote consultations (provisional in the sense that study design does not allow conclusions to be drawn across all clinical areas)

### Research Questions

This study will examine the following research questions:

1. What are the (often implicit or unspoken) communication strategies through which technology-mediated consultations for diabetes, cancer, and heart disease are successfully accomplished?
2. How do patients and clinicians address misunderstandings in technology-mediated consultations and what strategies are more effective?
3. What can we learn from detailed linguistic analysis of real-life remote consultations to guide other clinicians and patients interested in or actively using Skype and other social media?

### Overview of Study Design

NIHR and the Wellcome Trust separately funded studies to collect data on remote consultations with doctors and nurses. This study will combine multimodal data (video, audio, and screen captures at both “ends” of a remote consultation) from these 2 (independently conducted) studies involving up to 40 remote consultations and comparing these with a subset of up to 25 face-to-face consultations and analyze the interaction using techniques designed for fine-grained analyses of verbal and nonverbal interactions. This powerful technique has yet to be applied to remote consultations, partly because of the logistical difficulties of obtaining high-quality video and audio data at both ends of a consultation.

### Theoretical and Conceptual Framework

Findings from our own, and others, research highlight important interactional differences between remote and face-to-face consultations (see above) and indicate that the mode of communication can alter the interpersonal dynamics between patients and clinicians [10,18,19]. To examine the significance of this, we will make use of both long-established techniques developed for microanalysis of face-to-face and telephone conversations [73,74] and insights from recent work on mediated and multimodal interaction using both verbal and visual channels, for example, videoconferencing, vlogging, and courtroom video links [62,67]. We will use 2 complementary theoretical approaches that see communication as a dynamic interaction that emerges moment by moment and look beyond the traditional patient/clinician dyad to examine the role of technology in shaping interaction.

First, we will use the “ethnography of communication” (an approach that aims to produce systematic and richly contextualized descriptions of the communicative genres, events, and practices that are observed in a particular culture [75]) to identify the key features of remote consultations and attend systematically to the contextual factors (eg, lack of spatial proximity and restricted visual field) that may be producing differences with face-to-face consultations. Our focus will be on “communicative competence” [76] (ie, how participants in remote consultations deploy their tacit understanding of a particular communicative event and what competencies are needed to maximize the benefits of the encounter).

Second, we will use discourse analysis to guide fine-grained examination of the patterning of interaction at a “micro”-level (ie, how consultations are managed by participants moment by moment). Discourse analysis encompasses a number of approaches [77]. We will draw on concepts and techniques from several of these, including Conversation Analysis, which focuses on the resources used by participants in talk to create/maintain order and coherence [78-80], interactional sociolinguistics, which focuses on the use of context-specific frames and schemas to negotiate meaning in interaction [81], and multimodal discourse analysis, which focuses on the interaction of different modes and channels of communication, for example, verbal and visual, to produce meaning, especially in mediated environments [10].

### Setting

Data will be drawn from 2 independently conducted studies on remote consultations.

#### Setting 1

The VOCAL study (March 2015-July 2017) was undertaken with Barts Health, the UK’s largest acute trust. We studied 2 services, Diabetes and Pancreatic/Liver Cancer, both based in London boroughs characterized by high socioeconomic deprivation and ethnic and linguistic diversity. Barts Health is under pressure to deliver services more cost-effectively while responding to rising need and demand. Extending remote consultations is a part of that plan. The Diabetes Service (led by SV) has a tradition of ensuring that services are accessible and oriented to meeting the needs of the most vulnerable and serves a population with one of the UK’s highest prevalence of type 2 diabetes in the 16-25 age group. Engagement with traditional health service models is low. Remote consultations, where clinically appropriate, appear to be acceptable allowing for a flexible model of care. Experience delivering remote consultations since 2012 suggests they are popular with patients and staff.

The Royal London Hepato-Pancreato-Biliary Cancer Service (led by SB) is a tertiary service, which patients often travel long distances (up to 200 miles) to access. It provides contrasting demographic and clinical challenges to the diabetes example. Patients with pancreatic and liver cancer have a diverse demography but have in common a life-threatening diagnosis, major surgery, and a prolonged postoperative phase, in which they have to cope with multiple physical, emotional, and practical challenges. The service has been trialing remote consultations (largely for postoperative follow-up) since September 2015.

#### Setting 2

The OTQS study (on-going) is undertaken with the community-based, specialist nurse-led service funded by Oxford Health NHS Foundation Trust and working in liaison with the hospital-based heart failure service, local GPs, other community services (eg, palliative care nurses), integrated locality teams (occupational therapy, physiotherapy, mental health), social services, and 5 locality-based ambulatory assessment units providing emergency care for patients. The community heart failure nurses each have an active caseload of 100-120 patients, which they manage through a combination of community clinics, home visits, and telephone management. A high proportion of patients are unable to get to clinic (owing to frailty or fatigue) and home visits are time-consuming. Consequently, the remote option is a viable alternative. The team remains keen to evaluate whether remote consultations can help them deploy their limited resources safely, efficiently, and effectively without loss of patient or staff satisfaction. Some clinicians (particularly nurses) are skeptical because the functional and cognitive deficits in many patients with heart failure present a challenge to remote consulting.

### Cross Study Sample

We will include all of the remote consultations recorded in both studies. This currently gives a sample of 39 remote consultations (Table 1). We plan on collecting one more remote consultation in the heart failure service, raising the overall total to 40.

The goal of sampling has been to capture the breadth of (patients’ and staff’s) experience of remote consultation. The number of patients with cancer and heart disease is lower because there are greater practical and ethical challenges to gaining informed consent and avoiding harm, particularly in cases of heart failure which often require physical examination.

Within each subsample we have sought maximum variety in clinical, social, ethnic, and personal circumstances. In the diabetes service, we sought to include young adults, older people, limited English speakers, and women who recently had diabetes in pregnancy, all of whom, for various reasons, struggled to engage with the regular service. Our sample for cancer was drawn from a tertiary care surgical center and included those receiving postoperative follow-up and posttreatment surveillance. In the heart failure service, we sought patients with left ventricular systolic dysfunction or heart failure with preserved ejection fraction under the care of a cardiologist and those identified by the heart failure specialist nurses (tasked with educating newly diagnosed patients, up-titrating medications, and monitoring benefits or adverse effects of medication) as suitable for remote consultation. Further detail on sampling and the context in which consultations took place can be found in previous study reports [18,70]. Because remote consulting is a new medium with potentially harmful effects in some patients, patient participants were selected for invitation based on the clinician’s judgment from the denominator population of all those attending participating outpatient clinics. Exclusion criteria were no 3G access (VOCAL), or no 3G or Wi-Fi (OTQS) at home, lack of familiarity with the relevant technology, clinical inappropriateness (eg, need for physical examination), inability to give informed consent, and comorbidity preventing participation (eg, severe visual impairment).

To enable comparison, we collected 17 audio recordings of matched face-to-face consultations with patients diagnosed with diabetes, cancer, and heart failure. Additionally, we plan to collect a further 8 (giving a total of 25) face-to-face consultations, matched as closely as possible for the type of condition, type of appointment (eg, 6-month follow-up), patient demographics (eg, gender, ethnicity) and, in all cases, for the clinician.

### Description of Study Dataset

The core dataset of (currently 39, planned 40) video-recordings of remote consultations incorporates video, audio, and screen capture at both “ends,” clinician and patient, for each consultation along with detailed transcriptions.

Both studies captured 2 video streams, what the clinician sees and does in the clinic and what the patient sees and does at the remote site (the place where the patient consults from, typically, from the living room at home). To date, in 27 of the 39 consultations, we have recorded the clinician’s end of the consultation using a small digital camcorder. We used the same or equivalent technology for the patient end to capture video and good quality voice recordings. In each of the consultations, the camera’s field of view captures as much as possible of the individual and their orientation toward the screen (eg, a computer or tablet) as well as contextual detail in the room. This worked well in the “pilot phase” consultations. Once remote consultations became “business as usual,” it was harder for staff to find time to recruit participants and alert the research team for a planned consultation. This meant that 12 recordings were captured within the clinic but not on the patient end.

For personal/laptop computers, we used a commercially available screen capture software tool to record screen images showing on each party’s computer screen as a video file. We used an encrypted USB device to run this software on laptops/computers and positioned a second digital camera for tablets and mobile devices.

**Table 1:** Overview of cross study sample.

Illness	Total recorded	Male/female	Age range in years, median (SD)	Ethnicity (n)	Technology
Diabetes	18	5/13	21-50 (28)	White British (6); white other (2); black Caribbean (2); Asian Bangladeshi (2); Asian Indian (3); Asian other (3)	Skype using a personal computer (PC), laptop, tablet, or mobile phone device
Cancer	12	4/8	55-84 (74)	White British (8); white other (1); Asian Indian (1); black Caribbean (1)	Skype using PC, laptop, tablet, or mobile phone device
Heart failure	9	4/5	33-87 (76)	White British (9)	Skype or FaceTime using laptop, tablet or mobile phone device

We synchronized screen capture and video files into one using video editing software such that the video of the computer screen can be played exactly in parallel with a video of the patient looking at the screen. We then aligned the patient’s and clinician’s “ends” in a single editable file. These synchronized files allowed us to zoom in and slow down events to examine interactions, judgments, interpretations [82], the bodily conduct of (patient and clinician) participants, and the ways in which objects (eg, mobile devices and patient records) come to gain significance at particular moments [83]. We have also transcribed consultations using ELAN, a specialized program for transcribing and analyzing video and audio resources that has allowed us to capture verbal and nonverbal details of interactions and view these interactions repeatedly (a requirement of linguistic analysis [56,82]) and annotate audio and video streams at the level of a sentence, word, comment, or any other linguistic feature.

Ethnographic data, in the form of field notes from patients’ homes and each of the clinics, provides details of the patient’s domestic support, material circumstances, and cultural factors impacting on their self-management as well as the physical circumstances, under which the clinician makes the remote call, including the use of additional technologies (eg, electronic records).

### Analysis

Analysis is informed by ethnography of communication and discourse analysis (see above). Our focus is primarily on the (video-recorded) remote consultation data, which will be our starting point for the analysis. We will draw on the (audio-recorded) face-to-face consultation to explore the differences in talk across the 2 genres and on field notes to understand the clinical, organizational, material, and cultural contexts in which both face-to-face and remote consultations take place.

We will initially focus on any differences across all remote consultations, which will include exploring any differences in how the condition being investigated shapes the remote interaction, attending systematically to the contextual factors that may be producing any differences (eg, restricted visual field), the “communicative competence” [76] that participants in remote consultations deploy, and the competencies needed to maximize the benefits of the encounter. We will compare remote and face-to-face consultations to explore whether and how the affordances of the remote medium (ie, the way it constrains and enables interaction) change the interactional structure and content of the consultation and whether the spatial distance between participants, along with the fact that patients are somewhere other than the institutional space of the clinic, often their domestic space, alters the social and power relationships.

We will examine the patterning of interaction in remote consultations at a micro level, how consultations are managed by participants turn by turn and moment by moment using a range of discourse analytic techniques. On the basis of work done so far [18], the issues we think are likely to repay close analysis include the following: opening sequences (which have been shown to work differently in video environments [64,84]), the management of turn-taking (which may be affected by the way technology constrains participants’ visual orientation to each other and to relevant objects [62,85]), the use of back-channeling and other displays of acknowledgment/active listening (verbal and potentially nonverbal, eg, changes in head position [86]), repair (how participants deal with interactional problems, including those whose source is the remote location or the technology itself [87]), the use of questions (including whether/how patients and clinicians use them [88]), and the expression of stance and affect (particularly when clinicians need to communicate complicated or sensitive information or make requests/ask questions that might embarrass a patient).

### Project Management and Governance

The QuARC study will be based at the University of Oxford and include NHS partners in participating sites in Oxford and East London. The study is largely desk-based, involving a researcher with specialist experience in linguistics (LMS) bringing together and analyzing existing datasets. Meetings between team members will occur at least monthly by teleconference and 3-monthly face-to-face to share emerging findings and develop the analysis.

The program will be supported by an independently chaired, intersectoral steering group with representation from health services, policy makers, lay members, and external academics. We anticipate that this group will serve as an intersectoral discussion forum, a conduit to national policy, and a link with front-line clinical teams.

### Patient and Public Involvement and Engagement

Patients and their caregivers have been key to our research on remote consultations. We set up a dedicated patient advisory group (PAG) in 2015, the main purpose being to incorporate patient feedback within our work. Patients have reviewed key documents and fed back experiences about remote consultation services. Members of PAG felt that all patients should be offered the remote consultation option so that services would be available to all patients who chose it. This view was strongly and universally held. Implicit was the assumption that all patients, and clinicians, are au fait and confident with the technology and are able to manage (potentially very different) ways of communicating online. This insight informed our decision to develop guidance to support patients and clinicians when communicating online (see below).

### Ethics

Approval for VOCAL and OTQS studies was gained from National Research Ethics Service Committee London–City Road and Hampstead (REC reference: 14/LO/1883) on 2014 Dec 8 and South Central–Berkshire Research Ethics Committee (15/SC/053), respectively. All participating staff and patients in both studies gave their informed consent to be audio- and video-recorded during consultations and for data to be used for research purposes.

## Results

We seek to place detailed, granular descriptions of communication in technology-mediated consultations in the public domain. We believe that the emerging field of remote consultations will benefit from our research (particularly given the current sparsity of high-quality qualitative studies) and that our methodology may be taken up and applied by others interested in the interactional detail of remote consultations.

Our plan for dissemination is as much about contributions to the process as it is about end outputs [89]. Hence, an important feature of the QuARC study will be the level of collective engagement by different stakeholders in the unfolding project. We already have a network of policy makers (eg, NHS Digital), NHS Trusts (currently over 50), and patient/caregiver groups (eg, Diabetes UK) interested in or already using remote consultations. Drawing on techniques successfully applied in health technology codesign [90,91], we will invite professional, clinical, and service user representatives from across these sites to a series of codesign workshops and use a mix of presentations, video extracts, and interactive tasks (eg, card prompts) combined with narrative-based approaches (eg, “storyboards”) to collaboratively develop draft guidance for clinicians and service users. Guidance will be refined and finalized remotely, producing resources for patients and clinicians that can support effective communication in remote consultations and help to avoid/resolve problems (eg, regarding the technology and how it shapes or constrains clinical aims and outcomes).

## Discussion

The QuARC study is intended to deepen our understanding of how remote consultations work (and what makes them work more or less well) and benefit patients and clinicians by offering practical guidance on maximizing the effectiveness of remote consultations and avoiding/resolving any problems associated with mediation, such as transactional problems which may interfere with the achievement of desired clinical aims and outcomes or interpersonal problems which may affect the clinician-patient relationship. There is already a significant body of research focused on communication and interaction in face-to-face consultations [48,50,52-55]. To our knowledge, this is the first study, in which fine-tuned microanalysis of interactions in remote consultations will be conducted. We will also compare interaction across remote and face-to-face consultations. The latter will necessarily be limited given that our face-to-face comparator data consists of audio-recorded (ie, verbal) and not video-recorded (ie, visual) data. The study deliberately focuses on a small number of consultations undertaken in 3 clinical services in the English NHS. We anticipate rich insights into the communicative utility of the remote genre; however, caution will be needed in considering relevance to other settings and conditions.

One of the key findings of our work on remote consultations to date has been that although some clinicians are very keen to use this format, others are reluctant or opposed. One major benefit of having written, agreed guidance for both patients and clinicians is that the more reluctant clinicians will (we anticipate) be more confident to try this approach themselves. The written guidance could form the basis of local or national short courses and be submitted to Royal Colleges for consideration and endorsement. In this way, we believe that we will support a steady increase the number of clinicians willing to use the new technology and support them to do so safely and appropriately. However, we offer a final note of caution. In our experience, both clinicians and patients come on board gradually. Some are early adopters, whereas others are (for various reasons) more reluctant. What we are hoping for is to “shift the bell curve” through the provision of systematic, evidence-based guidance, thus helping to normalize this new way of interacting.

## Abbreviations

DNA: did not attend
DREAMS: Diabetes, Review, Engagement and Management via Skype
NHS: National Health Service
NIHR: National Institute for Health Research
OTQS: Oxford Telehealth Qualitative Study
PAG: patients advisory group
QuARC: Qualitative Analysis of Remote Consultations
RCT: randomized controlled trial
VOCAL: Virtual Online Consultations-Advantages and Limitations
